# Microstructural Properties of Human Brain Revealed by Fractional Anisotropy Can Predict the After-Effect of Intermittent Theta Burst Stimulation

**DOI:** 10.1093/texcom/tgab065

**Published:** 2021-12-15

**Authors:** Ikko Kimura, Hiroki Oishi, Masamichi J Hayashi, Kaoru Amano

**Keywords:** DTI, fractional anisotropy, interindividual variability, iTBS, rTMS

## Abstract

Intermittent theta burst stimulation (iTBS) delivered by transcranial magnetic stimulation (TMS) produces a long-term potentiation-like after-effect useful for investigations of cortical function and of potential therapeutic value. However, the iTBS after-effect over the primary motor cortex (M1) as measured by changes in motor evoked potential (MEP) amplitude exhibits a largely unexplained variability across individuals. Here, we present evidence that individual differences in white matter (WM) and gray matter (GM) microstructural properties revealed by fractional anisotropy (FA) predict the magnitude of the iTBS-induced after-effect over M1. The MEP amplitude change in the early phase (5–10 min post-iTBS) was associated with FA values in WM tracts such as right superior longitudinal fasciculus and corpus callosum. By contrast, the MEP amplitude change in the late phase (15–30 min post-iTBS) was associated with FA in GM, primarily in right frontal cortex. These results suggest that the microstructural properties of regions connected directly or indirectly to the target region (M1) are crucial determinants of the iTBS after-effect. FA values indicative of these microstructural differences can predict the potential effectiveness of repetitive TMS for both investigational use and clinical application.

## Introduction

Repetitive transcranial magnetic stimulation (rTMS) is widely used to modulate cortical excitability for experimental investigations and for the treatment of diseases such as major depression, movement disorders, and chronic pain (Lefaucheur et al. 2020). Thus, it is of great experimental and clinical value to predict those subjects or patients most responsive prior to application. Intermittent theta burst stimulation (iTBS) is a rTMS protocol consisting of three pulses at 50 Hz repeated at 200-ms intervals (5 Hz) and delivered intermittently for 191 s (600 pulses in total) (Huang et al. 2005). This method is currently the focus of intensive preclinical and clinical investigations (Suppa et al. 2016; Rounis and Huang 2020), as this pattern can evoke a long-term potentiation (LTP)-like after-effect in corticospinal excitability lasting for around 30 min when applied over primary motor cortex (M1) (Huang et al. 2005; López-Alonso et al. 2014), significantly longer than conventional rTMS protocols (e.g., continuous 5 Hz rTMS) using the same stimulation length.

A major concern when using iTBS, however, is the substantial interindividual variability in the magnitude of this after-effect (Hamada et al. 2013; Hinder et al. 2014; López-Alonso et al. 2014; Corp et al. 2020; Leodori et al. 2021; Ozdemir et al. 2021). Thus, prior assessment of iTBS susceptibility would be useful for obtaining robust results in rTMS experiments (López-Alonso et al. 2014) and for identifying patients most likely to benefit from clinical application. Previous studies have found that age, genetic polymorphisms, time of the day iTBS is delivered, and hormone levels are associated with interindividual variability in the after-effect (for review, see Suppa et al. 2016, and for the result from the meta-analysis, see Corp et al. 2020). However, these factors are not region-specific, and the underlying neuromodulatory mechanisms critical for the iTBS after-effect are unknown.

Recently, Nettekoven and colleagues reported that the functional connectivity (FC) values between M1 and other cortical regions predicted the magnitude of the iTBS after-effect (Nettekoven et al. 2015). This result suggests that the strengths of neural connections with iTBS targets may also influence the interindividual variability. However, given that FC fluctuates depending on the subject’s state of mind and alertness (Rosenberg et al. 2016), anatomical connectivity, which is independent of these factors, may be a more robust predictor. Fractional anisotropy (FA), a metric of diffusion-weighted magnetic resonance imaging (dMRI) that quantifies the anisotropy in directionality of water diffusion, is associated with the microstructural properties of neural tissue such as cell density and the orientation, diameter, and myelination of axons (Le Bihan et al. 2001; Beaulieu 2002; Le Bihan 2003). Several studies have found that FA values can predict the ability to learn new motor skills (Tomassini et al. 2011; Schulz et al. 2015; Lehmann et al. 2019), recovery rate of motor function after stroke (Kumar et al. 2016; Puig et al. 2017; Soulard et al. 2020), and degree of behavioral change induced by rTMS (Vanbellingen et al. 2020), suggesting that FA is associated with synaptic plasticity within motor-associated regions.

We speculated that the microstructural properties reflected by dMRI are associated with interindividual differences in the after-effect of iTBS. To test this hypothesis, we examined whether regional FA values within the human brain, which reflect local microstructural properties of white matter (WM) and gray matter (GM), are correlated with interindividual variability in the iTBS after-effect over M1. We report that FA values in certain WM and GM regions predict the magnitude of the after-effect during the early and late phases, respectively, while other factors, such as sex and age, had little influence. These findings suggest that metrics derived from dMRI measurement, such as FA, are strong predictive indicators of regional responses to iTBS and possibly other rTMS protocols.

## Materials and Methods

**Figure 1:**
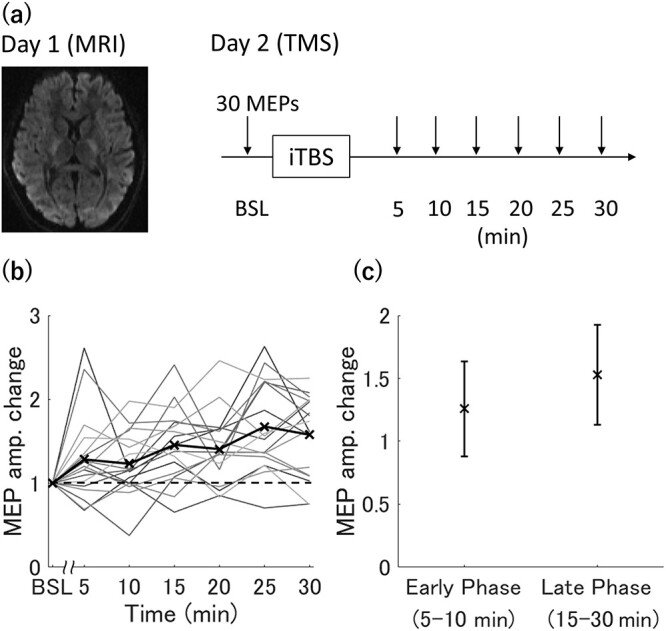
Overview of the experimental protocol for measuring changes in the MEP amplitude after iTBS over the primary motor cortex. (*a*) On day 1, MRI data were acquired. On day 2, a TMS experiment was performed in which MEPs were measured before and after iTBS. (*b*) Individual time courses of MEP amplitude changes for all participants (*n* = 18). The horizontal axis indicates the time after iTBS, while the vertical axis indicates the MEP amplitude normalized to baseline amplitude (BSL). Thick black line and cross indicate the mean change in MEP amplitude across individuals. (*c*) Changes in MEP amplitude during the early phase (5–10 min post-iTBS) and late phase (15–30 min post-iTBS). The black cross indicates the mean change and error bars indicate ±1 SD.

The experiment required 2 days for completion by each participant. On day 1, magnetic resonance imaging (MRI) data were collected. On day 2, the after-effect of iTBS was assessed by measuring the amplitude of motor evoked potentials (MEPs) pre- (baseline) and post-iTBS (Fig. 1*a*).

### Participants

Eighteen healthy adult volunteers (13 males and 5 females; age: 20–24 years; mean ± standard deviation [SD], 21.7 ± 1.0 years) participated in this study. All subjects were right-handed according to the Edinburgh handedness inventory (Oldfield 1971) and reported no history of neuropsychiatric diseases. The experiments were approved by the institutional ethics and safety committees of the National Institute of Information and Communications Technology and were performed in accordance with the Declaration of Helsinki. All participants provided informed consent after a full explanation of study protocols and aims.

### Transcranial Magnetic Stimulation

The iTBS was applied over the right M1 to target the left first dorsal interosseous (FDI) muscle. We note that the after-effect of iTBS showed no significant difference in MEP when iTBS was applied over contralateral M1 of the dominant versus nondominant hand (Suppa et al. 2008). Each session was performed in the afternoon, starting around 1 or 3 PM, to mitigate known diurnal variations in response (Suppa et al. 2016). To determine the optimal stimulator output for MEP measurement and iTBS, resting motor threshold (RMT) and active motor threshold (AMT) were defined for each participant prior to the iTBS session using the relative-frequency method (Rossini et al. 2015). The motor thresholds defined by this conventional method were nearly the same as those defined by the recently proposed adaptive-threshold hunting method (Ah Sen et al. 2017). The RMT was defined as the lowest intensity that evoked a MEP of at least 50 μV on 5 out of 10 trials in the left FDI muscle at rest (Rossini et al. 2015), while AMT was defined as the lowest intensity that evoked a MEP of at least 200 μV on 5 out of 10 trials in the left FDI muscle during volitional contraction at approximately 10% of maximum (Rossini et al. 2015). Thirty MEPs from the left FDI muscle were recorded approximately 10 min before iTBS (baseline) and for up to 60 min after iTBS at 5-min intervals, with the stimulator output set to 120% of the RMT. The stimulation was targeted to the hotspot over the right M1 evoking the strongest MEP in the left FDI muscle. Coil orientation was also optimized to elicit the largest MEPs, with the coil handle pointing backward and approximately 45° from the midline. Before iTBS, alertness of the participants was also assessed by the Stanford Sleepiness Scale (SSS) (Hoddes et al. 1972). All MEPs for determination of RMT and the iTBS after-effect were evoked using a monophasic Magstim 200^2^ stimulator (Magstim) with a figure-of-eight 70-mm standard coil, while AMT was determined using a biphasic Magstim Rapid^2^ stimulator (Magstim) with a figure-of-eight 70-mm air film coil.

The iTBS was delivered with the same stimulator and coil as used for AMT measurements. The iTBS protocol was the same as that introduced by Huang et al. (2005), consisting of 2-s trains of three pulses at 50 Hz repeated every 200 ms (5 Hz). Trains were repeated 20 times at an 8-s intertrain interval for 191 s (a total 600 pulses). The stimulation intensity was usually set to 80% of the AMT. However, when 80% AMT exceeded the system’s upper limit for TBS protocols (corresponding to 50% maximum stimulator output [MSO]), the stimulator output was set at this upper limit. The coil position and orientation during iTBS remained the same as the ones optimized for RMT measurements, following Huang’s original paper (Huang et al. 2005). The coil position was monitored and recorded using a Brainsight neuronavigation system (Rogue Research Inc.), and the mean deviation of coil position from the mean stimulated location across trials was calculated for each participant.

### Electromyography

MEPs from the left FDI muscle were recorded as surface electromyogram (EMG) signals using pregelled Ag-AgCl electrodes, with the active electrode placed on the muscle belly and the reference electrode placed on the metacarpophalangeal joint of the left index finger. The MEP signals were amplified and recorded with 16–470 Hz band-pass filtering and 3 kHz digitization using Brainsight (Rogue Research Inc.).

### MEP Analysis

MEP amplitudes were measured using custom software and the Veta-Toolbox (Jackson and Greenhouse 2019) implemented in Matlab (MathWorks). For three participants, we were unable to measure MEPs 35 min post-iTBS and later due to coil overheating, and hence, only the time points up to 30 min post-iTBS were used for subsequent analysis. At each time point, MEPs >2.5 SD from the mean of 30 trials were rejected as outliers (Fried et al. 2017) and the remaining MEP amplitudes were averaged and divided by the mean baseline MEP amplitude to calculate the after-effect of iTBS. Since previous studies have reported bimodal changes in MEP amplitude after iTBS, with a trough at 12.5 min post-iTBS (Huang et al. 2005), we separated the time points into two phases, early (5 and 10 min post-iTBS) and late (15–30 min post-iTBS) phases, and the change in amplitude at each time point within each phase relative to baseline was averaged. To validate this phase stratification, we performed hierarchical clustering analysis (Supplementary Fig. S2). First, similarity measures were calculated between each time point as the Euclidean distance between vectors of MEP amplitude for all participants and then clustering analysis was conducted using the single linkage method (Murtagh and Contreras 2012). We also performed a two-tailed one-sample Student’s *t*-test for the MEP amplitude changes in the early and late phases. In each phase, we confirmed that the MEP amplitude change was not affected by the outlier rejection method. In particular, the MEP amplitude changes were highly correlated between outlier rejection by raw data (0.018% rejected) and log-transformed data (0.015% rejected) (early phase, rho = 0.99, *P* < 0.001; late phase, rho = 0.99, *P* < 0.001)).

#### Image Acquisition

Prior to the iTBS session, dMRI data, with *b* = 1000 s/mm^2^ and *b* = 2000 s/mm^2^, were collected from each participant using a Siemens Vida 3T scanner and 64-channel array head coil (Siemens). Both diffusion-weighted images were obtained using a multislice 2D single-shot spin-echo echo-planar sequence with the following parameters: voxel size = 2 × 2 × 2 mm, matrix size = 106 × 106 × 74, iPAT reduction factor = 2, multiband acceleration factor = 3, phase-encoding direction = A-P, and time of repetition (TR) = 5300 ms. The number of directions and time to echo (TE) differed between the two datasets, with number of directions = 30 and TE = 71 ms for the *b* = 1000 s/mm^2^ dataset and with number of directions = 60 and TE = 86 ms for the *b* = 2000 s/mm^2^ dataset. Eleven nondiffusion-weighted (*b* = 0 s/mm^2^) images were also acquired to minimize EPI distortion, five images with the same TE as in the *b* = 1000 s/mm^2^ dataset with three images reversed phase-encoding directions (i.e., P-A), and three images with the same TE and phase-encoding directions (i.e., A-P) as in the *b* = 2000 s/mm^2^ dataset. Total acquisition time for dMRI was around 10 min for each participant.

For neuronavigation of TMS coil position and surface-based analysis, a T1-weighted MP-RAGE image was also obtained for each participant (voxel size = 1 × 1 × 1 mm, TE = 2.48 ms, TR = 1900 ms, flip angle = 9°).

#### Image Analysis

The dMRI data were preprocessed using tools from the FMRIB software library (FSL 6.0.1, https://fsl.fmrib.ox.ac.uk/fsl). The FSL topup tool was used to correct for EPI distortions due to inhomogeneity in the magnetic field (Andersson et al. 2003), and the eddy tool was used to correct for eddy current (Andersson and Sotiropoulos 2016) with outlier replacement and slice-to-volume correction (Andersson et al. 2016). Nonbrain tissue was removed using brain extraction tool (Smith 2002). FA was calculated for each *b* = 1000 s/mm^2^ and *b* = 2000 s/mm^2^ dataset using the DTIFIT application of FSL and was averaged across individual datasets to obtain individual FA maps.

To determine whether the difference in FA reflects the density and/or dispersion of orientation of neurites (Zhang et al. 2012), neurite density index (NDI) and orientation dispersion index (ODI) were calculated using the NODDI toolbox v1.0.3 (www.nitrc.org/projects/noddi_toolbox). NODDI considers the neurite compartment as a set of sticks, which restrict the diffusivity of water along their perpendicular direction. The density of neuritic compartments was defined as NDI, while the orientation dispersion of the neurites was defined as ODI. Because dendrites and axons are mainly located in the GM and WM, respectively, the NDI/ODI in GM and WMs were considered to provide microstructural indices for dendrites and axons, respectively (Zhang et al. 2012). Before fitting the NODDI model, diffusion-weighted images at each *b* value were divided by the mean nondiffusion-weighted image obtained with the same TE value to merge each dataset (Owen et al. 2014; Chang et al. 2015; Palacios et al. 2020). The fitting was performed using the default settings for WM, while the intrinsic free diffusivity parameter was changed to 1.1 × 10^−3^ mm^2^/s for GM (Fukutomi et al. 2018, 2019; Guerrero et al. 2019).

The tract-based spatial statistics (TBSS) tool of FSL (Smith et al. 2006) was used for whole-brain voxel-wise analysis of WM. First, the FA map for each participant was nonlinearly registered to 1 × 1 × 1 mm^3^ MNI152 space (McConnell Brain Imaging Centre, Montreal Neurological Institute). From the mean FA image across participants, a common skeleton was extracted to represent the main WM structure. This skeleton was thresholded at FA > 0.2 (default) and FA data warped to MNI152 space were then projected onto this skeleton. The NDI and ODI were also projected onto the skeleton using the same warp used for FA.

For surfaced-based analysis, FreeSurfer (Version 6.0.0, https://surfer.nmr.mgh.harvard.edu/) was used to obtain individual cortical surfaces (Fischl et al. 1999). After removing nonbrain tissues, the structural brain image was normalized into Talairach space and the intensity of each image was normalized. The normalized brain image was then segmented into GM, WM, and cerebral spinal fluid (CSF). The GM–WM boundary (WM surface) and GM–CSF boundary (pial surface) were used for surface reconstruction. Utilizing the folding pattern, the surface was registered to the standard surface space (fsaverage). All dMRI-derived GM metrics were sampled from the midpoints of white and pial surfaces as the partial-volume effect is less likely to impact the results (McNab et al. 2013). The sampled data was warped to fsaverage space and were smoothed with a Gaussian Kernel of 10-mm full width at half maximum (FWHM) across the cortical surface according to a previous study using FA for surface-based analysis (Stock et al. 2020).

#### Statistical Analysis

The FSL randomize tool was used to test the statistical significance of associations between each phase of MEP amplitude change and the TBSS data. For the surface-based analysis, the FSL permutation analysis of linear models tool was used to test these associations in GM. In both WM and GM, we performed 5000 permutation tests and employed threshold-free cluster enhancement (Smith and Nichols 2009). To specify locations significantly correlated with MEP amplitude change, significantly associated WM and GM voxel clusters were labeled using the Johns Hopkins University white-mater tractography atlas (https://fsl.fmrib.ox.ac.uk/fsl/fslwiki/Atlases) and Desikan-Killiany atlas (Desikan et al. 2006), respectively. To distinguish the microstructural properties contributing to interindividual variability in FA, we calculated Pearson’s correlation coefficients between mean FA and mean NDI or ODI in each cluster and tested the significance by 5000 permutation tests (Supplementary Figs. S4 and S5). In all analysis, Bonferroni correction was used to adjust for possible spurious findings due to multiple testing.

We also calculated Pearson’s correlation coefficients between all measured continuous variables and MEP amplitude change within each phase, while two-tailed unpaired *t*-tests were performed to assess how MEP amplitude change is affected by sex and iTBS intensity (80% of AMT or 50% of MSO). A *P* < 0.05 was considered to be significant for all tests. These analyses were performed using JASP (ver. 0.13.1 for Windows, https://jasp-stats.org/).

**Figure 2:**
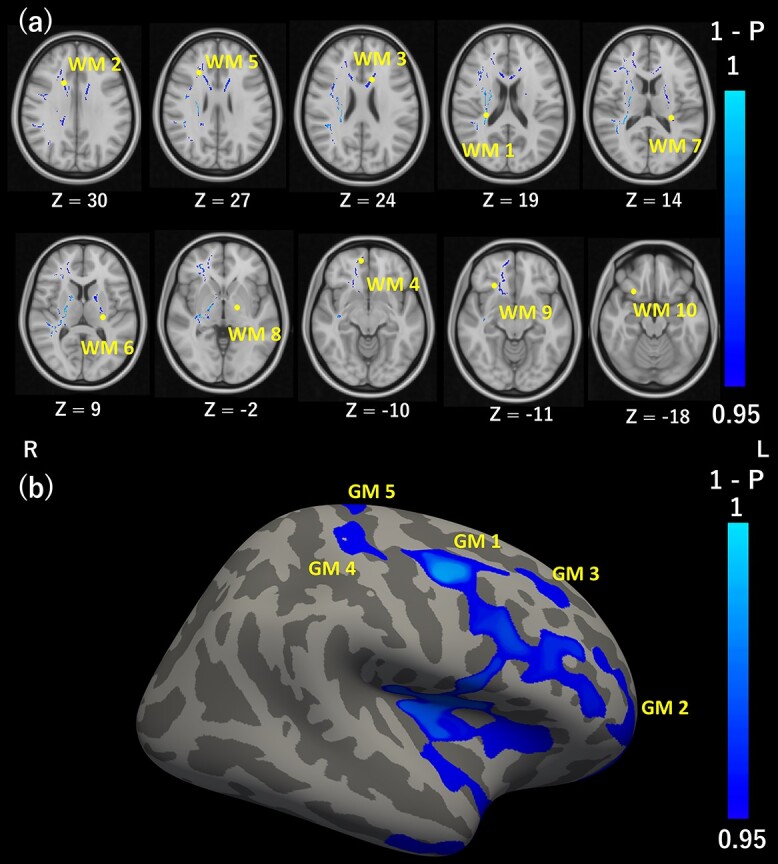
Correlations between regional FA and MEP amplitude changes during early and late phases. (*a*) TBSS analysis of significant correlations between FA in WM and early-phase MEP amplitude change. Clusters in blue represent voxels showing significant negative correlations with FA. The numbers (WM 1–10) correspond to individual voxel clusters. Yellow dots indicate the lowest *T*-values (strongest correlations) in each cluster. The axial slices are displayed according to radiological convention (left on the picture is right in the brain) with the range in MNI coordinates from *z* = 30 (top left) to *z* = −18 (bottom right). (*b*) Surface-based analysis of significant correlations between FA in GM and late-phase MEP amplitude change. Clusters in blue represent voxels showing significant negative correlations with FA. The numbers (GM 1–5) correspond to the individual voxel clusters.

## Results

All participants completed both MRI scans and iTBS experiments, and no adverse events occurred during these procedures. The mean deviation of the stimulated location from the mean stimulated location across trials was 0.85 mm (range: 0.33–1.64 mm) (see Supplementary Figs S1 for the error bar of the trial-by-trial deviation for each participant). Plotting the individual time courses of MEP amplitude changes post-iTBS relative to baseline revealed substantial interindividual variation (Fig. 1*b*) in accord with previous reports (Hamada et al. 2013; López-Alonso et al. 2014). Consistent with the previous study showing the two phases of MEP amplitude change after iTBS (Huang et al. 2005), our hierarchical clustering analysis revealed the similar separation in the time course (early phase; 5 and 10 min post-iTBS, late phase; 15–30 min post-iTBS) (Supplementary Fig. S2). Therefore, the relationships between the MEP amplitude change and brain microstructural properties were analyzed separately for the early and late phases. MEP amplitudes post-iTBS were significantly facilitated in both phases (early phase, *t* = 2.89, *P* = 0.020; late phase, *t* = 5.68, *P* < 0.001).

We tested the associations between regional FA and MEP amplitude change. During the early phase, MEP amplitude change was significantly and negatively correlated with FA of WM tracts anatomically connected to M1 (Fig. 2*a*; for the spatial maps in all slices, see Supplementary Fig. S3). These tracts include right superior longitudinal fasciculus, right posterior corona radiata, right internal capsule, and right corticospinal tract, constituting one large cluster (WM1 in Figure 2*a*). There were also significant negative correlations between MEP amplitude change and FA in bilateral corpus callosum (WM 2 and 3), right forceps minor (WM 4 and 5), left anterior limb of the internal capsule (WM 6), left retrolenticular part of the internal capsule (WM 7), left posterior limb of the internal capsule (WM 8), and right uncinate fasciculus (WM 9 and 10). Conversely, regional FA in the GM was not correlated with the early phase MEP amplitude change.

In contrast to the early phase of MEP amplitude change, the late-phase change was negatively correlated with FA exclusively in GM regions (Fig. 2*b*), including the right caudal middle frontal region, right pars opercularis, right insula, right superior temporal, and right middle temporal regions (GM 1). Negative correlations were also found between late-phase MEP amplitude change and FA in the anterior part of the right rostral middle frontal region (GM 2), dorsal part of the right rostral middle frontal region (GM 3), right postcentral gyrus (GM 4), and right precentral and postcentral gyrus (GM 5). There were no significant correlations between the late-phase MEP amplitude change and FA in the WM.

We also tested whether interindividual differences in MEP amplitude change during the early and late phases were associated with other collected measurements (Table 1). Age, handedness, and interval between the MRI scan and iTBS session were not significantly correlated with either early- or late-phase MEP amplitude change (*P* > 0.05). Similarly, stimulus intensity for MEP induction, iTBS stimulus intensity, SSS score, mean baseline MEP amplitude, and mean deviation of stimulated locations from the mean stimulated location across trials were not significantly correlated with the MEP amplitude change (*P* > 0.05). Unpaired *t*-test also revealed no significant differences in MEP amplitude change between sexes (early phase, *t* = 0.77, *P* = 0.45; late phase, *t* = 0.26, *P* = 0.80) or between subjects receiving iTBS at 80% of AMT or 50% of MSO (early phase, *t* = 1.27, *P* = 0.33; late phase, *t* = −0.23, *P* = 0.82). 

Finally, to identify the factors contributing to FA values in each voxel cluster associated with MEP amplitude change, we investigated the correlations between mean FA and mean NDI or ODI, which, respectively, reflect neurite density or the dispersion of the orientation of neurite within a voxel (Zhang et al. 2012). The FA was positively correlated with NDI in WM 1 (right superior longitudinal fasciculus and right corticospinal tract), WM 4 and 5 (right forceps minor), and WM 6 (left anterior limb of internal capsule) (Supplementary Fig. S4a), while FA was negatively correlated with ODI in all WM clusters (Supplementary Fig. S4b). The FA values of GM 1–3 (right frontal regions, right insula, and right temporal regions) were also negatively correlated with ODI (Supplementary Fig. S5a), while FA values of GM 4–5 (the right postcentral gyrus) were positively correlated with NDI (Supplementary Fig. S5b).

## Discussion

In this study, we demonstrated multiple significant associations between MEP amplitude changes after iTBS over M1 (the iTBS-induced after-effect) and the microstructural properties of GM and WM regions associated with M1. No other measured factors showed significant associations. Thus, individual variation in these microstructural properties can explain, at least in part, the known individual variation in iTBS after-effect, thereby providing a potential method to predict responsive individuals prior to neuroscientific investigations and possibly iTBS-based therapy.

The early-phase MEP amplitude change was negatively correlated with regional FA values in WM tracts, including right superior longitudinal fasciculus, corpus callosum, right forceps minor, left internal capsule, and right uncinate fasciculus. By contrast, the late-phase MEP amplitude change was negatively correlated with regional FA values in GM areas such as right frontal cortex, right insula, right temporal cortex, and right postcentral gyrus. Collectively, these findings might suggest that distinct mechanisms underlie the early and late phases of the iTBS after-effect.

**Table 1:** Correlations between MEP amplitude changes and other measured factors

Factors	MEP amplitude change (early phase)		MEP amplitude change (late phase)	
	$\rho$	$P$	$\rho$	$P$
Age Handedness Visit interval RMT MEP stimulus intensity iTBS stimulus intensity SSS Baseline MEP amplitude The mean deviation of stimulated location	−0.10 0.27 0.07 −0.31 −0.22 −0.13 0.05 −0.07 −0.15	0.68 0.27 0.79 0.21 0.39 0.62 0.85 0.78 0.54	−0.00 0.23 −0.13 −0.09 −0.10 −0.01 −0.19 −0.35 0.00	1.00 0.37 0.60 0.72 0.71 0.97 0.45 0.16 1.00	

Note: MEP, motor evoked potential; RMT, resting motor threshold; MEP stimulus intensity, stimulus intensity to induce MEP; iTBS intensity, stimulus intensity to perform intermittent theta burst stimulation; SSS, stanford sleepiness scale; Baseline MEP amplitude, mean size of MEP amplitude before iTBS (baseline).

### Significant Increase in MEP Amplitude Post-iTBS

We noted significant and continuous increase in the MEP amplitude until 25 min post-iTBS. By contrast, Hamada and colleagues reported no significant facilitation in the MEP amplitude after iTBS (Hamada et al. 2013). A recent meta-analysis investigating the after-effect of iTBS also showed that the increase of MEP amplitude attenuated 20–30 min post-iTBS (Corp et al. 2020). Given that MEP amplitude measured 25 min post-iTBS previously showed a sustained facilitation for young participants (18–28 years), but returned to baseline for older participants (65–76 years) (Dickins et al. 2015), the apparent discrepancy between our results and those of previous studies (Hamada et al. 2013; Corp et al. 2020) may be due to the difference in participants’ age. In the studies by Hamada et al. and Corp et al., the mean ages were 30.3 and 41.9 years, respectively, whereas participants in the present study were all in the range of young participants (20–24 years, mean age: 21.7 years).

### Negative Correlations of Regional WM FA with Early-Phase MEP Amplitude Change

The early-phase MEP amplitude change was negatively correlated with regional FA values in WM tracts related to motor function, including the right corticospinal tract (WM 1), right superior longitudinal fasciculus (WM 1), and corpus callosum (WM 2 and 3), which are functionally and anatomically connected to M1 (Catani and de Schotten 2012). The corticospinal tract sends output from M1 to the contralateral spinal cord and ultimately to distal muscles (Lemon 2008) and the output strength (number of motor neurons recruited) determines MEP amplitude (Bestmann and Krakauer 2015). The superior longitudinal fasciculus is the main intrahemispheric tract connecting frontal areas (e.g., premotor cortex, dorsolateral prefrontal cortex [DLPFC], and M1) and parietal areas (e.g., angular gyrus and supramarginal gyrus), and is crucial for motor planning, motor imagery, and visuo-motor tasks (Nakajima et al. 2020). The corpus callosum connects the bilateral M1 (Hofer and Frahm 2006) and mediates both interhemispheric inhibition (Ferbert et al. 1992) and facilitation (Hanajima et al. 2001).

The FA values within a part of the corpus callosum that connects bilateral frontal regions (WM 2 and 3), right forceps minor (WM 4, 5, 9, and 10), and left internal capsule (WM 6–8) were also negatively correlated with MEP amplitude change. Given that these tracts are not directly connected to the right M1, their microstructural properties may be similar to those of the WM connecting to the right M1. The similarity is possibly mediated by factors affecting the general WM functional properties, such as the gene polymorphisms of the brain-derived neurotrophic factor, which was known to influence the microstructural properties of WM (Chiang et al. 2011).

Further examination of the associations between regional FA values in WM and both NDI and ODI, which, respectively, reflect the density and the dispersion of orientation of neurites within a voxel (Zhang et al. 2012), provided clues to the nature of these microstructural differences underlying individual variation in iTBS after-effect. A larger MEP change was associated with lower FA in multiple tracts, and in several of these tracts (including right superior longitudinal fasciculus and right corticospinal tract of WM 1, right forceps minor in WM 4 and 5, and left anterior limb of internal capsule in WM 6), FA was negatively correlated with NDI (Supplementary Fig. S4a), while FA was negatively correlated with ODI in all clusters (Supplementary Fig. S4b). Therefore, lower FA values in WM 1, 4, 5, 6, and 10 may reflect less consistent fiber orientation (higher dispersion), and a smaller neurite fraction, possibly reflecting smaller diameter of neural fibers or lower myelination. Alternatively, lower FA values in the other WM clusters may reflect only less consistent fiber orientation.

### Negative Correlations of Regional GM FA with Late-Phase MEP Amplitude Change

In contrast to the early phase, the late-phase MEP amplitude change was negatively correlated with regional GM FA, primarily in the right frontal cortex (GM 1–3). These regions include right premotor cortex (GM 1), right DLPFC (GM 1), and right anterior prefrontal cortex (aPFC) (GM 2), all of which are implicated in motor function. The premotor cortex is crucial for motor planning and transferring that information to M1 (Hoshi and Tanji 2007), while the DLPFC integrates inputs from multiple sensory modalities to decide on the action to take (Yarrow et al. 2009) and the aPFC is important for motor response inhibition such as in go/no-go tasks (Boecker et al. 2007; Wriessnegger et al. 2012). Both the premotor cortex (Civardi et al. 2001; Koch et al. 2007; Bäumer et al. 2009; Groppa et al. 2012) and DLPFC (Hasan et al. 2013; Cao et al. 2018) modulate the activity of ipsilateral M1. Furthermore, premotor cortex was shown to modulate the plasticity of ipsilateral M1 (Huang et al. 2018).

The late-phase MEP amplitude change was also negatively correlated with FA in the right postcentral gyrus (GM 4 and 5) and right insula (GM 1). The postcentral gyrus provides sensory feedback to M1 (Kaelin-Lang et al. 2002) and M1 modulates activity of the postcentral gyrus (Katayama and Rothwell 2007), suggesting reciprocal functional connections. The insula is a part of the saliency network and facilitates motor responses indirectly via the anterior cingulate cortex (Menon and Uddin 2010).

The FA values of GM 1–3 (right frontal regions, right insula, and right temporal regions) were also negatively correlated with ODI (Supplementary Fig. S5b), indicating that the greater MEP amplitude change associated with lower FA may reflect more complex dendritic branching (Zhang et al. 2012). By contrast, the FA values of GM 4–5 (the right postcentral gyrus) were positively correlated with NDI (Supplementary Fig. S5a), suggesting that a larger late-phase after-effect may be facilitated by a lower density of apical dendrites (Zhang et al. 2012; Ball et al. 2013). Therefore, we speculate that the structure of dendrites in the GM might affect the late-phase MEP amplitude change.

### Microstructural Properties May Influence the After-Effect of iTBS

We found that the microstructural properties of non-M1 regions correlated with the iTBS after-effect. In addition to M1, non-M1 regions structurally connected to M1 were previously shown to regulate the MEP amplitudes. For example, the MEP amplitudes were affected by activities not only in M1 located contralateral to the stimulated side (Dickins et al. 2015) but also in ipsilateral prefrontal regions, such as premotor cortex and DLPFC, connected to the stimulated M1 (Civardi et al. 2001; Koch et al. 2007; Bäumer et al. 2009; Groppa et al. 2012; Hasan et al. 2013; Cao et al. 2018). Moreover, the activities of prefrontal regions were shown to modulate the iTBS after-effect on ipsilateral M1 (Huang et al. 2018). These studies suggest that the activities of non-M1 regions contribute to determine the iTBS after-effect on M1. However, its relationship to the microstructural properties of WM and GM is still open question.

Besides the contribution of non-M1 regions, several neural mechanisms might explain the distinct early- and late-phase iTBS after-effects. First, short-term potentiation is reflected more in the early phase than in the late phase (Ugawa 2012). Second, gene expression associated with the LTP of excitatory and inhibitory interneurons were facilitated 10–20 min after iTBS (i.e., during the early phase), while that of inhibitory interneurons was suppressed 20–40 min after iTBS (i.e., during the late phase) (Hoppenrath and Funke 2013). While these results suggest the involvement of different mechanisms in the early- and late-phases of MEP amplitude changes, how they, respectively, relate to the WM and GM structures remains unclear. Future studies are warranted to clarify the physiological mechanisms which underlie the relationship between microstructural properties and the MEP amplitude changes.

One might also ask how the iTBS after-effect is coupled with the functional and structural changes in remote regions. The after-effect on remote regions can be assessed with structural (Jung and Lambon Ralph 2021) and functional MRI (Nettekoven et al. 2014; Jung and Lambon Ralph 2016). It would be appealing to investigate the relationship between structural and functional changes measured by MRI and the iTBS after-effect. Future studies are also necessary to compare the MEP and MRI measurements between pre- and post-iTBS and to investigate its relationship.

Surprisingly, in the current study, the FA in M1 was not predictive of the MEP amplitude change. Substantial differences in the anatomical structure between M1 and other cortical areas might explain the significant correlations of FA in remote regions contrary to M1. First, layer V in M1 is much thicker than that in other cortical areas (Brodmann 1909). Second, the giant pyramidal neurons named “giant Bets cells” were exclusively localized to the layer V of M1 (Lassek 1941). Because of these unique properties of M1, we speculate that the restriction of water determining the FA values in M1 mainly reflects a microstructural property of the somata of layer V neurons. By contrast, the restriction of water determining FA values in the prefrontal regions would reflect the property of dendrites, structures which are mainly found across layers. Future neuroimaging studies with a higher spatial resolution are needed to determine whether the microstructural properties of the GM in M1 predict the magnitude of iTBS after-effect.

### Limitations

Potential limitations of this study include the use of FA to evaluate the microstructural properties of GM, as FA may not be a sensitive indicator of GM properties due to the relatively large partial-volume effect (Aggarwal et al. 2015). To resolve this issue, we analyzed FA only in the middle part of the GM (Stock et al. 2020) or along the major structures of the WM (Smith et al. 2006), which we believe helped to minimize the impact of the partial-volume effect. Furthermore, previous dMRI studies with higher spatial resolution have demonstrated the validity of FA for quantifying and distinguishing the structures of cortical layers (McNab et al. 2013; Aggarwal et al. 2015). Nonetheless, we cannot rule out the possibility that the analyzed regions are affected by partial-volume effects from surrounding areas. Future neuroimaging studies with higher spatial resolution are needed to clarify how microstructural properties in regions associated with M1 influence the after-effect following iTBS.

Another limitation of our study is we stimulated the right M1 instead of the left M1, contrary to previous studies investigating the after-effect of iTBS on M1 (Corp et al. 2020). Nevertheless, several iTBS studies targeted the right M1 to assess the plasticity of M1 (Suppa et al. 2008; Platz et al. 2018; Ding et al. 2021). Furthermore, Suppa and colleagues reported that performing iTBS over M1 of the dominant or nondominant hemisphere did not significantly affect the MEP amplitude change on the contralateral FDI muscle (Suppa et al. 2008). This finding suggests that stimulating the right instead of left M1 in the current study would not affect the MEP amplitude changes.

## Conclusion

The microstructural properties of certain WM regions are negatively associated with the magnitude of the early-phase iTBS after-effect, while the microstructural properties of certain GM regions are negatively associated with the magnitude of the late-phase iTBS after-effect. These results suggest that FA measured by dMRI can be a powerful tool for prediction of experimental and therapeutic iTBS responses.

## Funding

This work was supported by the Japan Society for the Promotion of Science (Grants-in-Aid for Scientific Research JP18H05523 to K.A. and JP18H01101 to M.J.H., Grant-in-Aid for Scientific Research on Innovative Areas JP19H05313 to M.J.H., and Grants-in-Aid for JSPS Research Fellow JP20J11101 to H.O.) and Japan Science and Technology Agency (PRESTO JPMJPR17J1 to K.A. and PRESTO JPMJPR19J8 to M.J.H).

## Notes

We thank Ian Greenhouse for advice on EMG data analysis and Tomoya Kawashima for critical comments on the manuscript. *Conflict of Interest*: The authors declare no conflicts of interest associated with this manuscript.

## Supplementary Material

KimuraI_CerebralCortexCommunications_Supplementary_20211122_tgab065
